# Comparison of Heart Rate Variability, QTc, and JT Interval Between Diabetic Patients and Healthy Controls: Role of Gender and Phases of Menstrual Cycle

**DOI:** 10.7759/cureus.24179

**Published:** 2022-04-16

**Authors:** Kandan Balamurugesan, Shanmugavel Karthik, Jean Fredrick

**Affiliations:** 1 Medicine, Jawaharlal Institute of Postgraduate Medical Education & Research, Puducherry, IND; 2 Physiology, Jawaharlal Institute of Postgraduate Medical Education & Research, Puducherry, IND; 3 Physiology, All India Institute of Medical Sciences, Kalyani, Kalyani, IND

**Keywords:** qtc interval, menstrual cycle, jt interval, heart rate variability, diabetes mellitus type 2

## Abstract

Background and objectives

Type 2 Diabetes mellitus (T2DM) is a heterogeneous group of metabolic disorders with variable degrees of insulin resistance and altered glucose metabolism. Increased attention in studying the role of gonadal hormones in diabetes is not only due to their relation to insulin sensitivity, and glucose tolerance but also to the gender-specific nature of the prevalence of various diabetic complications. The cyclical change in the hormone level in females will make it necessary to consider the menstrual cycle while analyzing the risk factors for diabetes. Hence, the role of gender and menstrual cycle in T2DM are analyzed here using the simple non-invasive cardiovascular risk indices like heart rate variability (HRV), QT interval corrected for heart rate (QTc), and JT interval.

Materials and methods

In this analytical study, T2DM patients in the age group of 18-45 years with less than five years duration from diagnosis and taking not more than two anti-hyperglycemic drugs were included. Time and frequency domains of HRV analysis, QTc, and JT intervals were compared with age and BMI matched control group. The comparison of these parameters was also made between two genders in the diabetic group and they were analyzed across different phases of the menstrual cycle in female diabetic patients when physiological variation in the gonadal hormones occurred as a natural phenomenon.

Results

HRV parameters were reduced and the QTc and JT intervals were prolonged in diabetic patients of both genders. Reduction in low-frequency (LF) band power and high-frequency (HF) band power of HRV analysis in diabetic females were statistically significant in the luteal phase of the menstrual cycle in comparison with age and BMI-matched healthy controls. There was no significant difference in the HRV parameters, QTc, and JT interval between the male and female diabetic groups. HF band power is significantly reduced in the menstrual phase and relatively higher in the follicular phase when compared to the luteal phase among female diabetic patients.

Conclusion

The reduced sympathetic and parasympathetic activity were observed in diabetic patients of both genders and they were significant in the luteal phase of diabetic females compared to the healthy control group. Vagal activity is relatively higher in the follicular phase of the menstrual cycle in female diabetic patients.

## Introduction

The prevalence of type 2 diabetes mellitus (T2DM) is increasing worldwide. In India, it has risen from 7.1% in 2009 to 8.9% in 2019. Age-adjusted comparative prevalence of diabetes is expected to reach 10.8 by 2045 [1-3]. The onset and severity of the complications in T2DM depend on the ethnicity, gender, age, and duration of the diabetic state [4,5]. Cardiac complications are more common than other microvascular complications of diabetes mellitus in the Asian-Indian population [5].

Though the prevalence of diabetes is less in females, the relative risk of coronary heart disease and stroke in females are 40% and 20% higher, respectively, compared to male diabetic patients [5,6]. However, in the healthy population, the cardioprotective effect in females during their reproductive life is said to be due to the gonadal hormones, estrogen and progesterone. It is evident with many studies reporting the association of estrogen with heart rate variability (HRV), baroreflex sensitivity, and insulin sensitivity [7-9].

Estrogen acts on ventricular muscle fibers and cardiac nodal tissues and modifies the cardiac chronotropic and inotropic actions through long-acting calcium channels (ICa_L_) and potassium channels (IKs). Progesterone and testosterone differ from estrogen in these actions [9-11]. It is also reported that estrogen regulates the glycaemic level through two types of receptors. ERα protects β-cell survival, whereas ERβ reduces ERα function and provokes β-cell apoptosis [12,9]. In vivo, under normal conditions, progesterone suppresses gluconeogenesis following plasma insulin induction in a mouse model. However, progesterone increases blood glucose via gluconeogenesis in both insulin-deficient and insulin-resistant mice models [13]. Hyperglycemia is increasingly recognized as an independent risk factor for cardiovascular disease in diabetes [14,15]. Accordingly, gonadal hormones influence cardiac function directly [9-11] and indirectly through modifying the autonomic nervous system [16,17] and glycemic index [12,13], especially in diabetes.

HRV and QT interval corrected for heart rate (QTc) are simple and non-invasive markers in predicting cardiovascular risk [18-21]. Albeit conflicting reports, many studies have noted that HRV and QTc are different in various phases of the menstrual cycle due to the variation in the levels of gonadal hormones [22-26]. JT interval is a significant independent predictor of incident coronary heart disease events [27]. There is a paucity of information regarding the role of gender and the different phases of the menstrual cycle on cardiac autonomic modulation in T2DM patients when the level of endogenous gonadal hormones is varied.

## Materials and methods

This study was conducted in the Department of Physiology, Indira Gandhi Medical College and Research Institute (IGMC&RI), Puducherry, India, from July 2012 to June 2013 after obtaining approval from the institute's research and ethics committees (IEC/IGMC/Apvl/2012 dated June 8, 2012). Participants in the age group of 18-45 years with body mass index (BMI) less than 25 were included in this study. Patients with T2DM for less than five years duration and who were taking not more than two oral anti-hyperglycemic drugs participated in this study. The female participants in both the control and diabetes groups had regular menstrual cycles (28-32 days). The readings from the female participants were taken three times in the menstrual cycle. The first and second recordings were recorded on the 9th or 10th day (follicular phase) and the 21st or 22nd day (luteal phase) of a menstrual cycle. The third recording was obtained on the third or fourth day of the next cycle (menstrual phase).

 Thirty-four patients with T2DM (20 male and 14 female participants) were included in the study group. In the control group, 19 apparently healthy participants (12 male, 7 female) were included. Participants with a history of ischemic heart disease, hypertension, chronic renal disease, other endocrine disorders, chronic medication and hormonal therapy, and menstrual disorders were excluded from the control group. Diabetic patients who were on insulin therapy were also excluded from this study. After 15 minutes of complete rest in the supine position, ECG recordings were taken for five minutes with spontaneous breathing using the INCO data acquisition system (Instruments & Chemicals Pvt. Ltd., Haryana, India, and Niviqure Meditech Pvt. Ltd, Karnataka, India), in a room temperature maintained at 26°C between 10 am to 11.30 am. The data were saved in American Standard Code for Information Interchange (ASCII) format and analyzed for power spectral analysis of HRV using the software Kubios HRV Version 2.1 (Kubios, Kuopio, Finland), Niviqure software analysis version 52.0 (Niviqure Meditech Pvt. Ltd, Karnataka, India), Microsoft Office 2007 (Microsoft Corporation, Redmond, Washington, United States) were used to estimate QTc (using Bazett formula) [27] and corrected JT interval (JTc) (JTc = QTc - QRS duration) [28]. Taskforce guidelines and American Hospital Association (AHA)/American College of Cardiology Foundation (ACCF)/Heart Rhythm Society (HRS) recommendations for the standardization and interpretation of the ECG were followed during the analysis [29].

Time-domain and frequency domain analyses of heart rate variability were done with mean heart rate (bpm), root mean square of successive differences between normal heartbeats (RMSSD) (ms) and total power (ms^2^), low-frequency band power (LF) (ms^2^), high-frequency band power (HF) (ms^2^), LF (normalized unit (nu)), HF (nu), LF/HF ratio, respectively. These parameters were compared between diabetic and control groups of both genders. In female participants, comparisons were made in three phases of the menstrual cycle separately between the control and diabetic population. Gender difference and variation in different phases of the menstrual cycle in HRV, QTc, and JT interval parameters were also analyzed in T2DM.

Data were analyzed using JASP 0.16.1 software, 2022. Continuous variables such as anthropometry parameters, mean heart rate, RMSSD, total power, LF, HF, LF/HF ratio, LF (nu) and HF (nu) from the HRV analysis, QTc, and JT interval were analyzed with Shapiro-Wilk test to determine the distribution of data. Parametric data were represented as mean and standard deviation (SD). Non-parametric data were represented as the median and interquartile range (IQR). 

Comparison of HRV parameters, QT, and JTc intervals between males and each phase of the menstrual cycle among female participants in both the control and the diabetic group were done using the Mann-Whitney test. Comparison of HRV parameters, QTc, and JT intervals between three phases of the menstrual cycle among diabetic women was done using Friedman’s test. A post hoc comparison was done using Conover’s post hoc test. A p-value less than 0.05 was considered statistically significant.

## Results

Table 1 shows the demographic characteristics of the population studied. There was no significant difference in age (p=0.177) between the control and diabetic groups (Table 1). BMI was also similar between the diabetic and control groups (p=0.39).

**Table 1:** Demographic details of all the participants (Mean ± SD) Comparisons between the control and diabetic group for age and BMI were done using unpaired t-test. Chi-square test was used to compare the gender difference between the groups. p-value <0.05 was considered as the level of significance.

S.No		Control Male	Control Female	Diabetic Male	Diabetic Female	p-Value
1	Age (Years)	30.18 ± 2.6	32.35 ± 2.23	31.38 ± 2.2	33.68 ± 3.21	0.177
2	Gender (n)	12	7	20	14	0.91
3	BMI (Kg/m^2^)	22.86 ± 1.67	23.52 ± 3.78	23.55 ± 2.32	24.54 ± 1.85	0.39

Time-domain analysis of HRV showed a significant reduction in RMSSD (p value=0.029) in diabetic males (Table 2). Mean heart rate was higher in male diabetics without statistical significance. Among the frequency domain parameters, total power was significantly lower (p 0.024) in male diabetic patients. Both LF and HF in HRV were reduced in diabetic patients. LF (nu), and LF/HF ratio were reduced, and HF (nu) was increased in the diabetic population. QTc, JT intervals were prolonged (Table 2) in patients with T2DM without statistical significance.

**Table 2:** Comparison of heart rate variability, QTc, and JT intervals between control and T2DM groups in male participants (Median (IQR)) Data were presented as median (interquartile range). Data were analyzed using the Mann-Whitney U test. *p-value <0.05 was considered statistically significant. Mean HR: mean heart rate in beats per minute; RMSSD: root mean square of successive differences between normal heartbeats; VLF: very low frequency; LF: low frequency; HF: high frequency; LF (nu): low frequency power in the normalized unit; HF (nu): high frequency power in the normalized unit; QTc: corrected QT interval; T2DM: type 2 diabetes mellitus

S No	Parameter	Control Male (n=12)	Diabetic Male (n=20)	P Value
1	Mean HR (bpm)	70.99 (20.10)	75.30 (12.100)	0.216
2	RMSSD (ms)	26.4 (16.8)	19.15 (11.57)	0.029*
3	Total Power (ms^2^)	1537.00 (1488.50)	664.50 (769.75)	0.024*
4	VLF Power (ms^2^)	833.00 (1029.00)	263.00 (244.00)	0.031*
5	LF Power (ms^2^)	400.50 (779.25)	172.00 (240.00)	0.100
6	HF Power (ms^2^)	301.00 (261.75)	163.00 (241.75)	0.123
7	LF (nu)	69.45 (31.27)	54.75 (20.77)	0.373
8	HF (nu)	29.85 (30.82)	45.10 (20.97)	0.350
9	LF/HF	2.38 (2.551)	1.22 (1.018)	0.322
10	QTc (s)	0.364 (0.040)	0.385 (0.037)	0.183
11	JT (s)	0.229 (0.055)	0.224 (0.030)	0.878

Comparison of HRV, QTc, and JT intervals were done between diabetic and healthy control in the respective phase of the menstrual cycle (Table 3). Total power, very low frequency power (VLF) band, HF, and LF were significantly reduced in the luteal phase with statistical significance with p-values 0.031, 0.012, 0.033, and 0.031, respectively.

**Table 3:** Comparison of heart rate variability, QTc, JT intervals between control and T2DM in female participants in three phases of the menstrual cycle. Data were presented as median (interquartile range). Comparison of each phase of menstrual cycle between control and T2DM female participants was done by Mann-Whitney U test. p-value <0.05 was considered statistically significant. Mean HR: mean heart rate in beats per minute; RMSSD: root mean square of successive differences between normal heartbeats; VLF: very low frequency; LF: low frequency; HF: high frequency; LF (nu): low frequency power in the normalized unit; HF (nu): high-frequency power in the normalized unit; QTc: corrected QT interval; JT: JT interval; T2DM: type 2 diabetes mellitus

	Control female paticipants			T2DM female participants						
	Menstrual Phase (n=7)	Follicular Phase (n=7)	Luteal Phase (n=7)	Menstrual Phase (n=14)	Follicular Phase (n=14)	Luteal Phase (n=14)	Control MP vs T2DM MP p -value	Control FP Vs T2DM FP p -value	Control LP Vs T2DM LP p -value	
Mean HR (bpm)	81.32(22.58)	84.64 (12.94)	83.13 (11.96)	81.92(15.66)	75.53 (13.16)	81.27(15.90)	0.938	0.173	0.813	
RMSSD (ms)	20.90(10.50)	16.60 (16.60)	22.40 (15.00)	16.10(19.12)	26.55(18.27)	17.10(8.35)	0.585	0.799	0.079	
Total Power (ms^2^)	509.00(534.50)	964.00 (1240.00)	1148.00(986.00)	391.00(430.00)	583.00(576.25)	411.00(144.50)	0.428	0.535	0.031*	
VLF Power (ms^2^)	210.00 (150.50)	368.00 (662.00)	513.00(419.50)	141.00 (152.00)	191.00(246.00)	217.50(164.25)	0.502	0.856	0.012*	
LF Power (ms^2^)	172.00(111.00)	187.00 (347.00)	356.00(381.00)	102.00(102.25)	128.00(226.25)	73.50(84.75)	0.391	0.488	0.031*	
HF Power (ms^2^)	86.00(299.00)	223.00 (190.000	279.00(357.00)	66.50(197.00)	181.00(223.75)	82.00(114.50)	0.360	0.488	0.033*	
LF (nu)	56.10(25.60)	53.00 (6.70)	53.00(8.65)	69.30(31.03)	62.35(27.55)	52.30(33.95)	0.126	0.577	0.971	
HF (nu)	43.90(23.550	46.30(6.80)	44.00(8.85)	30.70(30.95)	37.60 (27.70)	47.55(32.15)	0.126	0.533	0.971	
LF/HF	1.28(1.19)	1.14 (0.32)	1.20(0.42)	1.48(3.59)	1.67(1.38)	1.12(1.98)	0.178	0.015	0.971	
QTc (s)	0.37(0.04)	0.38 (0.04)	0.39(0.05)	0.394(0.03)	0.39 (0.03)	0.39(0.03)	0.370	0.737	0.799	
JT (s)	0.224(0.020)	0.21(0.02)	0.23(0.02)	0.25(0.02)	0.25(0.04)	0.24(0.02)	0.149	0.224	0.636	

Table 4 showed the comparison of HRV, QTc, and JT intervals between male and female diabetic patients in various phases of their menstrual cycle. Though none of the parameters were statistically significant, total power in HRV was recorded more in male diabetic patients. QTc and JT intervals were found to be relatively higher in female participants than male diabetic patients.

**Table 4:** Comparison of HRV, QTc, JT intervals in T2DM male participants and female participants in three phases of the menstrual cycle Data were presented as median (interquartile range). Comparison between male T2DM participants and each phase of female T2DM participants was done using the Mann-Whitney U test. P<0.05 was considered statistically significant Mean HR: mean heart rate in beats per minute, RMSSD: root mean square of successive differences between normal heartbeats; VLF: very low frequency; LF: low frequency; HF: high frequency; LF (nu): low frequency power in the normalized unit; HF (nu): high-frequency power in the normalized unit; QTc: corrected QT interval; JT: JT interval; HRV: heart rate variability; T2DM: type 2 diabetes mellitus

	Male T2DM N=15	Female T2DM					
		Luteal Phase (n=14)	Follicular Phase (n=14)	Menstrual Phase (n=14)	Male T2DM Vs Female T2DM LP	Male T2DM Vs Female T2DM FP	Male T2DM Vs Female T2DM MP
Mean HR (bpm)	75.30 (12.100)	81.27 (15.90)	75.53 (13.16)	81.92 (15.66)	0.396	0.717	0.461
RMSSD (ms)	19.15 (11.57)	17.10 (8.35)	26.55 (18.27)	16.10 (19.12)	0.753	0.336	0.753
Total Power (ms^2^)	664.50 (769.75)	411.00 (144.50)	583.00 (576.25)	391.00 (430.00)	0.138	0.877	0.181
VLF Power (ms^2^)	263.00 (244.00)	217.50 (164.25)	191.00 (246.00)	141.00 (152.00)	0.377	0.691	0.100
LF Powe r(ms^2^)	172.00 (240.00)	73.50 (84.75)	128.00 (226.25)	102.00 (102.25)	0.148	0.769	0.201
HF Power (ms^2^)	163.00 (241.75)	82.00 (114.50)	181.00 (223.75)	66.50 (197.00)	0.183	0.820	0.189
LF (nu)	54.75 (20.77)	52.30 (33.95)	62.35 (27.55)	69.30 (31.03)	1.000	0.717	0.104
HF (nu)	45.10 (20.97)	47.55 (32.15)	37.60 (27.70)	30.70 (30.95)	1.000	0.717	0.104
LF/HF	1.22 (1.018)	1.12 (1.98)	1.67 (1.38)	1.48 (3.59)	0.986	0.717	0.191
QTc (s)	0.385 (0.037)	0.39 (0.03)	0.39 (0.03)	0.394 (0.03)	0.529	0.637	0.545
JT Interval (s)	0.224 (0.030)	0.24 (0.02)	0.25 (0.04)	0.25 (0.02)	0.111	1.00	0.066

Time-domain and frequency domain analysis of HRV did not differ significantly among three phases of the menstrual cycle in female diabetic patients (Table 5) except HF band (p value 0.004). RMSSD in the time domain and total power, LF, and LF/HF were relatively higher in the follicular phase compared to the other two phases of the menstrual cycle.

**Table 5:** Comparison of heart rate variability, QTc, JT intervals in female T2DM patients in three phases of the menstrual cycle Data were presented as median (interquartile range). Data were analyzed using Friedman’s test to compare different phases of the menstrual cycle within the T2DM female participants. A post hoc test was done using Conover’s post hoc test. * P< 0.05 was considered statistically significant. + Significantly different from Menstrual Phase. ++ Significantly different from Follicular phase Mean HR: mean heart rate in beats per minute, RMSSD: root mean square of successive differences between normal heartbeats, VLF: very low frequency; LF: low frequency; HF: high frequency; LF (nu): low frequency power in the normalized unit; HF (nu): high frequency power in the normalized unit; QTc: corrected QT interval; JT: JT interval; T2DM type 2 diabetes mellitus

Female TSDM	Menstrual Phase (n=14)	Follicular Phase (n=14)	Luteal Phase (n=14)	p-value
Mean HR (bpm)	81.92 (15.66)	75.35 (13.16)	81.27 (15.90)	0.584
RMSSD (ms)	16.10 (19.12)	26.55 (18.27)	17.10 (8.350)	0.607
Total Power (ms^2^)	391.00 (430.00)	583.00 (576.25)	411.00 (144.50)	0.223
VLF Power(ms^2^)	141.00 (152.00)	191.00 (246.00)	217.50 (164.25)	0.395
LF Power(ms^2^)	102.00 (102.25)	128.00 (226.25)	73.50 (84.75)	0.395
HF Power (ms^2^)	66.50 (197.00)	181.00 (223.75)^+ ^	82.00 (114.50)^++^	0.004*
LF (nu)	69.30 (31.025)	62.35 (27.55)	52.30 (33.95)	0.145
HF (nu)	30.70 (30.95)	37.60 (27.78)	47.50 (32.15)	0.145
LF/HF	1.49 (3.60)	1.67 (1.38)	1.120 (1.978)	0.232
QTc (s)	0.39 (0.03)	0.39(0.026)	0.39(0.027)	0.257
JT (s)	0.25 (0.02)	0.25 (0.036)	0.24 (0.023)	0.191

## Discussion

There is no significant difference in age, gender, and BMI between the control and diabetic population in the current study.

HRV, QTc, and JT intervals in male diabetics

In the time domain analysis of HRV, RMSSD is significantly reduced (p value=0.028) in diabetic males (Table 2). Total power in the frequency domain of HRV is also found to be lower (p = 0.025) in male diabetic patients. It is similar to the findings reported by Burger et al. [30] and Ildiko et al. [31]. It is suggestive of reduced parasympathetic activity in the diabetic male participants. It is also supported by the increased resting heart rate and reduced HF in the diabetes group compared to the control group with similar age and BMI, though they are not statistically significant.

LF (0.04-0.15 Hz), LF (nu), and LF/HF ratio in HRV are reduced in male diabetic patients. These findings indicate that the sympathetic tone is also reduced in diabetic patients. It is out of line with the reports from Huggett et al. [32] and Perin et al. [33] as they have identified increased sympathetic tone in T2DM. It could be due to the inclusion of hypertension in their study population. In the current study, only diabetic patients who did not have any complications participated. It is also striking that reduced vagal activity is more distinct than sympathetic dysfunction in male diabetics.

VLF band is significantly reduced in male diabetes patients (p = 0.031). VLF band (0.0033-0.04 Hz) is mainly contributed by the heart’s intrinsic nervous system and the amplitude and frequency of its oscillations are influenced by the sympathetic nervous system (SNS) [34]. Parasympathetic nervous system (PNS), physical activity, thermoregulatory, renin-angiotensin, and endothelial influences on the heart are also associated with its generation [34]. Though the VLF band is best monitored over 24-hour recording, a five-minute sample does have about 12 complete periods of oscillation [34]. As the VLF band is influenced by SNS and PNS, a significant reduction of the VLF band in diabetic patients also indicates the dysfunction of both limbs of the autonomic nervous system (ANS), sympathetic and parasympathetic, in the diabetic group.

QTc is prolonged (Table 2) in male diabetes patients without statistical significance. Even though few studies reported low evidence of QTc prolongation in diabetes [34,35], many studies have highlighted the significant positive correlation between insulin resistance and QTc [36-39]. The difference in the JT interval, which is considered as a more appropriate measure of ventricular repolarization [40], is trivial between the control and diabetic groups.

HRV, QTc, JT intervals in female diabetics

Unlike the male gender, considering the menstrual cycle is important in the analysis of HRV and cardiac electrical activities in female diabetic patients, as there is a periodic fluctuation of gonadal hormones that will influence the cardiac repolarization and autonomic regulation.

To the best of our knowledge, comparison of HRV, QTc, and JT interval parameters in various phases of the menstrual cycle in the diabetic population are reported here for the first time, though the gender differences in diabetes [4,41,42] and the role of menstrual cycle on HRV and ECG intervals [22,23,25,26,43-45] are described in several studies. In our study, female diabetes during all three phases (menses, follicular, and luteal phases) show a reduction in HRV in many time-domain and frequency-domain parameters (Table 3).

Reduction in RMSSD, total power, LF, HF (nu), relatively increased resting heart rate, and LF/HF ratio is seen during the menstrual phase in the diabetic females when compared to healthy control (Table 3). QTc and JT intervals are prolonged in the diabetic female during the menstrual phase. Even though these differences are not statistically significant, it is noteworthy that similar observation is seen in the comparison between diabetic and healthy males (Table 2). In the initial few days of the menstrual cycle when female gonadal hormones are at low levels, autonomic dysfunction is similar to the male diabetic population. An increase in LF (nu) and LF/HF ratio could be the result of relatively more reduction manifested in the parasympathetic function.

In the follicular phase, observations of HRV, QTc, JT intervals (Table 3) and their comparisons with the control group are similar to the menstrual phase except in the reduction of heart rate and relatively increased RMSSD (a marker of vagal activity) noted in the diabetic female on HRV analysis. It could be due to the vagotonic and sympatholytic property of estrogen [46] as this hormone level is physiologically higher during the proliferative phase. Moreover, the sex steroid hormone-binding globulin (SHBG) - T2DM relationship is an independent factor in deciding the sex hormones levels in both men and women [47], Metabolic dysfunction with hyperglycemia, insulin resistance, and associated hyperinsulinemia in the early stages of T2DM [48] suppress the SHBG level [47], which will eventually increase the sex hormones level, could be the reason for the more prominent estrogen-mediated vagotonic responses observed in the diabetic population compared to the control female in their proliferative phase. As most of the HRV indices of vagal and sympathetic activities are reduced in this phase of the menstrual cycle, it is apparent that estrogen levels in the follicular phase of a diabetic female cannot provide respite for the negative sequel of diabetes on ANS.

Significant reduction in total power (p = 0.031), absolute power in the VLF (p = 0.012), LF (p = 0.031), HF bands (p = 0.033) of HRV are the sticking features in the female diabetics (Table 3) when compared to the healthy control during their luteal phase. Inhibitory action of progesterone through gamma-aminobutyric acid (GABA)-A receptors in various central autonomic nuclei might be the reason behind the significant reduction in heart rate variability [49]. It is tempting to report that there is some inkling of prominent autonomic dysfunction in the luteal phase involving both sympathetic and parasympathetic divisions while comparing the three phases of the menstrual cycle in female diabetic patients.

In all three phases of the menstrual cycle, QTc and JT intervals are prolonged (Table 3) in diabetic females. This change could be due to the result of the hyperglycemic state in diabetes and its consequence in the form of free radical [50] (reactive oxygen species (ROS)) damage. ROS increases the intracellular calcium in cardiac fibers and prolongs the repolarization of cardiac myocytes by influencing the nitric oxide level [50]. Sympatho-vagal imbalance resulting in ventricular electrical instability [50] could be another mechanism in the lengthening of QTc and JT intervals in both genders of the diabetic population. 

Gender difference in HRV, QTc, and JT intervals in T2DM

Though none of the HRV parameters, QTc, and JT intervals are significantly different (Table 4), resting heart rate is lower and total power in HRV is higher in male diabetic patients compared to female diabetics. It gives the impression of relatively more parasympathetic dysfunction in female diabetic patients except in the follicular phase. Increased RMSSD in the time domain and the increased HF band in frequency domain analysis of HRV are suggestive of the role of estrogen in deciding the net sympathovagal balance and its impact on heart rate variability.

QTc and JT intervals are found to be prolonged in female participants than male diabetic patients. Oxidative stress and its consequence on nitric oxide level results in prolonged repolarization of the cardiac myocytes. In general, females appear to be less susceptible to oxidative stress due to the antioxidant properties of estrogen and gender differences in NADPH‐oxidase activity [51]. It should also be noted that the gender difference in oxidative stress varies in ethnic groups and chronic diseases [51,52]. Prolonged QTc and JT intervals in the female diabetic group may be due to high oxidative stress and sympathovagal imbalance.

Duration of diabetes is less than five years in our study population. Even in such early stages of diabetes, though differences are statistically not significant, higher sympathetic tone and reduced vagal activity observed in female diabetic participants as opposed to males, could be the reason for the more diabetic complications associated with the female gender.

HRV, QTc, and JT intervals in various phases of the menstrual cycle in diabetic females

Time-domain and frequency domain analysis of HRV did not differ significantly in all parameters among three phases of the menstrual cycle in female diabetic patients (Table 5). Vagotonic effects of estrogen in the follicular phase are evident from the increased RMSSD, total power, and HF band (p-value 0.004) in the HRV analysis when compared with the other two phases of the menstrual cycle. However, the sympathetic activity, which is reflected in the LF, LF (nu), and LF/HF ratio, is not significantly different between the follicular phase and the other two phases.

Unlike the follicular phase, reduced vagal tone in the form of reduced RMSSD, HF (nu), and HF power in HRV analysis may be the result of a low level of estrogen and its influence on the ANS in the luteal phase. Spectral power in the LF, LF (nu), and LF/HF are relatively lower in the luteal phase compared to the follicular phase of the menstrual cycle. It could be due to the higher level of progesterone (with low sex hormone-binding globulin (SHBG)), which suppresses catecholamine secretion and sympathetic outflow [17,53]. It is also supported by the findings of Schmalenberger et al. [49,54], who have reported the central action of progesterone (P4) on the central autonomic network (CAN) primarily at the insular cortex and amygdala by the non-genomic pathway involving GABA-A receptor through its neuroactive metabolites allopregnanolone (ALLO) and pregnanolone.

Even though both sympathetic and parasympathetic dysfunctions are observed in the diabetic female population, the vagotonic effect of estrogen in the follicular phase and sympathetic suppression in the luteal phase are protrusive in the results.

## Conclusions

Autonomic dysfunction affecting both the sympathetic and parasympathetic systems with prolonged QTc and JT intervals is observed in both genders of the diabetic population. Primarily vagal dysfunction is observed in male diabetic patients. In female diabetic patients, autonomic dysfunction is more evident in the luteal phase affecting both sympathetic and parasympathetic components of ANS. This autonomic dysfunction with prolonged QTc and JT intervals during the luteal phase could predict increased cardiovascular risk among the various phases of the menstrual cycle in diabetic female participants. Further study in a larger population with subgroup analysis will give better insight into the pathophysiological basis of autonomic dysfunctions in the diabetic population.
